# Risk stratification of cirrhotic patients undergoing esophagectomy for esophageal cancer: A single-centre experience

**DOI:** 10.1371/journal.pone.0265093

**Published:** 2022-03-09

**Authors:** Julia K. Grass, Natalie Küsters, Marius Kemper, Jan Tintrup, Felix Piecha, Jakob R. Izbicki, Daniel Perez, Nathaniel Melling, Maximilian Bockhorn, Matthias Reeh

**Affiliations:** 1 Department of General, Visceral and Thoracic Surgery, University Medical Center Hamburg-Eppendorf, Hamburg, Germany; 2 I. Department of Medicine, University Medical Center Hamburg-Eppendorf, Hamburg, Germany; 3 German Center for Infection Research (DZIF), Partner Site Hamburg-Lübeck-Borstel-Riems, Hamburg, Germany; 4 Department of General and Visceral Surgery, University Medical Center Oldenburg, Oldenburg, Germany; School of Digestive & Liver Diseases, Institute of Post Graduate Medical Education & Research, INDIA

## Abstract

**Background:**

Concomitant liver cirrhosis is a crucial risk factor for major surgeries. However, only few data are available concerning cirrhotic patients requiring esophagectomy for malignant disease.

**Methods:**

From a prospectively maintained database of esophageal cancer patients, who underwent curative esophagectomy between 01/2012 and 01/2016, patients with concomitant liver cirrhosis (liver-cirrhotic patients, LCP) were compared to non-liver-cirrhotic patients (NLCP).

**Results:**

Of 170 patients, 14 cirrhotic patients with predominately low MELD scores (≤ 9, 64.3%) were identified. Perioperative outcome was significantly worse for LCP, as proofed by 30-day (57.1% vs. 7.7, p<0.001) and 90-day mortality (64.3% vs. 9.6%, p<0.001), anastomotic leakage rate (64.3 vs. 22.3%, p = 0.002) and sepsis (57.1 vs. 21.5%, p = 0.006). Even after adjustment for age, gender, comorbidities, and surgical approach, LCP revealed higher odds for 30-day and 90-day mortality compared to NLCP. Moreover, 5-year survival analysis showed a significantly poorer long-term outcome of LCP (p = 0.023). For risk stratification, none of the common cirrhosis scores proved prognostic impact, whereas components as Bilirubin (auROC 94.4%), INR (auROC = 90.0%), and preoperative ascites (p = 0.038) correlated significantly with the perioperative outcome.

**Conclusion:**

Curative esophagectomy for cirrhotic patients is associated with a dismal prognosis and should be evaluated critically. While MELD and Child score failed to predict perioperative mortality, Bilirubin and INR proofed excellent prognostic capacity in this cohort.

## Introduction

Ranking as the sixth leading cause of cancer-related mortality, esophageal cancer (EC) continues to be among the most aggressive tumours with limited prognosis [1], which can be altered most favourably by surgical intervention. Esophagectomy with radical lymphadenectomy represents the mainstay of curative therapy for EC. By continuously refined diagnostics and multimodal treatment, 5-year survival increased to 20–45% for surgically treated patients [2–4]. However, esophagectomy is associated with high rates of perioperative morbidity and mortality, which are significantly increased with comorbidities and patients’ age and adversely impact long-term outcomes [5–7].

Due to rising incidence and optimized therapeutical strategies resulting in longer survival, liver cirrhosis represents a common cause of morbidity [8]. Accompanied with a higher incidence of extrahepatic malignancies compared to non-cirrhotic patients [9], oncological therapy for liver cirrhotic patients (LCP) gains increasing relevance. As EC and cirrhosis share certain risk factors [10], LCP are reported to be exposed to an eight-fold elevated risk for the development of EC [9]. Though, liver cirrhosis depicts a crucial risk factor for non-hepatic surgery [11–13], with elevated morbidity and mortality following esophagectomy of 39.7–83.3% and 26.0–50.0%, respectively [14–18]. Although cirrhosis significantly compromises live expectancy [19], long-term survival after esophagectomy for EC has been reported to be comparable between LCP and non-liver-cirrhotic patients (NLCP) by several case series [14,16,18].

Limited evidence is available for treatment stratification of EC in LCP, in particular in terms of general health assessment and severity of liver cirrhosis, as stated by recent meta-analyses [20–22]. Thus, this study aims to evaluate, what factors might affect the perioperative and long-term outcome and whether radical esophagectomy represents a feasible option in the oncological treatment of cirrhotic patients.

## Methods

Patients with concomitant liver cirrhosis were identified from a prospectively maintained database of EC patients, who underwent esophagectomy at the University of Hamburg Medical Institutions between January 2012 to May 2016. The study was approved by the Ethics Committee Hamburg and conducted in accordance with the Declaration of Helsinki. According to local laws, no informed patient consent or statement by the federal ethics committee is required since the study is non-interventional and retrospective (§12HmbKHG—city law Hamburg). The study protocol was registered at clinicaltrial.gov (NCT04809870 on 18/03/2021). Informed consent was obtained from all patients included. The diagnosis of cirrhosis was confirmed by clinical signs, imaging, and histological proof, in particular in all cases of intraoperatively diagnosed cirrhosis. The severity of cirrhosis at the time of surgery was determined by preoperative laboratory data at hospital admission and preoperative imaging. Cirrhotic Patients with insufficient data for calculating preoperative model for end-stage liver disease (MELD) and Child-Turcotte-Pugh (CTP) scores were excluded from further analysis. Studied variables for both, cirrhotic and non-cirrhotic patients included gender, age, coexisting medical conditions, the ASA classification, and CCI, tumor stage administered by America Joint Committee on Cancer Union, 7^th^ edition, tumor localization, history of neoadjuvant therapy, laboratory data (creatinine, albumin, platelet count, bilirubin, and INR) and surgical approach. Moreover, for cirrhotic patients, etiology of cirrhosis, presence, and severity of preoperative ascites, portal hypertension, and esophageal varices were collected. Portal hypertension was defined by platelet count lower than 100 mrd/l and presence of ascites, splenomegaly, or esophageal varices.

Surgeries were performed as thoracoabdominal esophagectomy with either two-field lymphadenectomy and intrathoracic anastomosis (Ivor-Lewis) or cervico-thoracoabdominal esophagectomy with three-field lymphadenectomy and cervical anastomosis (McKeown) depending on the tumor localization. Reconstruction was conducted by gastric conduit. Eligible approaches were conventionally open, hybrid (abdominal laparoscopically and thoracic open), and completely minimally invasive. The stomach was checked for portal gastropathy, which was not present in this case series. The perioperative outcome was investigated by occurrence and severity of complications according to Clavien-Dindo classification, 30- and 90-day mortality as well as 5-year survival analysis. Pulmonary and cardiac complications are defined by any organ-related major complications (Clavien-Dindo ≥ 3). Anastomotic leakage was defined as any endoscopically proven anastomotic dehiscence and Chyle leak was defined by a concentration of triglycerides (TG) in drain fluids ≥ 3 times TGs in serum.

Data management and statistical analysis were performed using IBM SPSS Statistics for Macintosh, Version 25.0. (Armonk, NY: IBM Corp.). For univariate analyses, the Student *t* test was applied for parametric continuous variables and the Man-Whitney-U test for nonparametric continuous variables. Categorical variables were tested using χ^2^-Test or the Fisher exact test as appropriate. A generalized linear model was used for adjusted outcome analysis, adjusting for age, gender, surgical approach, and comorbidity index. The sensitivity and specificity of available scores and parameters were calculated using receiver operating characteristics (ROC) curves. Survival rates were estimated using the log-rank test and described by Kaplan-Meyer curves. A two-sided p-value < 0.05 was considered as significant. The study was conformed to the standards of the Declaration of Helsinki.

## Results

### Clinicopathological parameter

From the prospective database of 170 patients, who underwent esophagectomy for esophageal cancer between 01/2012 and 04/2016, 14 patients with concomitant cirrhosis could be identified (Table 1). Both cohorts were of comparable age (64.4 ± 8.7 vs. 63.4 ± 10.8, p = 0.826) while gender distribution differed, with significantly more female patients among the LCP (50.0% vs. 14.1%, p = 0.003). Both, American Society of Anaesthesiology (ASA) and Charlson Comorbidity Index (CCI) demonstrated a significantly poorer general condition of LCP (p<0.001 and p = 0.004, respectively). Nonetheless, NLCP also suffered from relevant comorbidities, reflected by 52.6% CCI of three or more points. Histology, grading, tumor size and location, nodal status, metastatic status, UICC stage, and proportion of neoadjuvant therapy were comparable between both groups. Surgical procedures were equally distributed with cervico-thoracoabdominal esophagectomies in 21.3% and 20.0%, respectively (p = 0.953). Significantly more LCP were operated using minimally invasive techniques compared to NLCP (21.3% vs. 10.2%, p = 0.017). Two of those suffered from a CTP A cirrhosis, one had a Child B cirrhosis. All minimally invasive operated patients had an elevated CCI from 5–6 and a small tumor burden (UICC I).

**Table 1:** Clinicopathological parameter of cirrhotic and non-cirrhotic patients.

	LCP	NLCP	p-value
	14	156	
Age	64.1±8.7	63.4±10.8	0.826
Gender			**0.003**
female	7 (50.0)	22 (14.1)	
male	7 (50.0)	134 (85.9)	
ASA			**<0.001**
1	1 (7.1)	50 (32.1)	
2	1 (7.1)	74 (47.4)	
3	11 (78.6)	32 (20.5)	
4	1 (7.1)	0 (0.0)	
Charlson Comorbidity Index	3.9±1.5	2.6 ± 1.6	**0.004**
0	0 (0.0)	16 (10.3)	0.105
1	1 (7.1)	27 (17.3)	
2	2 (14.3)	31 (19.9)	
+3	11 (78.6)	82 (52.6)	
Histology			0.318
AC	6 (42.9)	95 (60.9)	
SCC	8 (57.1)	61 (39.1)	
Grading			0.895
no grading after neoadjuvant therapy	2 (14.3)	24 (15.4)	
G1	2 (14.3)	12 (7.7)	
G2	5 (35.7)	70 (44.9)	
G3	5 (35.7)	49 (31.4)	
G4	0 (0.0)	1 (0.6)	
pT			0.600
pT0	2 (14.3)	17 (10.9)	
pT1	6 (42.9)	41 (26.3)	
pT2	2 (14.3)	20 (12.8)	
pT3	4 (28.6)	75 (48.1)	
pT4	0 (0.0)	3 (1.9)	
pN			0.107
pN0	10 (71.4)	86 (55.1)	
pN1	0 (0.0)	30 (19.2)	
pN2	4 (28.6)	24 (15.4)	
pN3	0 (0.0)	16 (10.3)	
M			0.604
M0	14 (100.0)	143 (91.7)	
M1	0 (0.0)	13 (8.3)	
UICC			0.317
IA + IB	8 (57.1)	58 (37.2)	
IIA + IIB	3 (21.3)	40 (25.6)	
IIIA + IIIB	3 (21.3)	58 (37.2)	
Localization			0.448
upper third	1 (7.1)	8 (5.1)	
middle third	5 (35.7)	25 (16.0)	
lower third	8 (57.1)	123 (78.8)	
Neoadjuvant Therapy (%)	6 (42.9)	55 (35.5)	0.575
Procedure			1.000
thoracoabdominal (Ivor-Lewis)	11 (78.6)	20 (80.0)	
cervico-thoracoabdominal (McKeown)	3 (21.3)	5 (20.0)	
Technique			**0.046**
conventional open	11 (78.6)	140 (89.7)	
minimally invasive	3 (21.3)	8 (5.1)	
Hybrid abdominal minimally invasive, thoracic open (Ivor Lewis)	0 (0.0)	8 (5.1)	

Numbers are presented as mean ± standard deviation or absolute numbers and percentages. p-values in bold indicate statistical significance between cohorts. ASA: American Society of Anaesthesiologists, LCP: Patients with concomitant liver cirrhosis, NLCP: Patients without concomitant liver cirrhosis, UICC–Union internationale contre le cancer, y–years.

### Severity of liver cirrhosis

64.3% of LCP presented with a preoperative MELD score ≤ 9, 28.6% with MELD score 10–13, and 7.1% with a MELD score > 13, according to 28.6% CTP A and 71.4% CTP B patients (Table 2). Seven patients had a preoperatively diagnosed liver cirrhosis, 57.1% with a MELD score ≤ 9, three of these patients were prepared by a preoperative transjugular intrahepatic portosystemic shunt (TIPS). MELD (p = 0.577) and CTG scores (p = 1.000) were not significantly different between patients with a pre- and intraoperative diagnosis of cirrhosis. Portal hypertension was present in 35.7% of LCP. The most common etiology of cirrhosis was alcohol abuse (85.7%). Moreover, 64.3% of LPC suffered from severe hypalbuminaemia (< 25mg/dl), which did not significantly differ from the NLCP cohort (50.4%, p = 0.385).

**Table 2:** Liver-specific clinicopathological parameter of cirrhotic patients.

	LCP (n = 14)
MELD Score	9.5±3.5
≤ 9	9 (64.3)
10–13	4 (28.6)
>13	1 (7.1)
CTP Pugh	7.0±1.1
A	4 (28.6)
B	10 (71.4)
TIPS preop	3 (21.4)
Liver cirrhosis diagnosed preop	7 (50.0)
MELD ≤ 9	4 (57.1)
MELD > 9	3 (42.9)
Esophageal varices	6 (42.6)
Portal hypertension	5 (35.7)
Etiology of LC	
ethyl toxic	12 (85.7)
HBV / HCV	1 (7.1)
unclear	1 (7.1)
Hypalbuminaemia	
≥ 35 mg/dl	2 (14.3)
34–25 mg/dl	3 (21.4)
< 25 mg/dl	9 (64.3)
Ascites preop	5 (35.7)
Splenomegaly	4 (28.6)

All numbers are indicated as absolute numbers and percentages or mean ± standard deviation.

HBV: Hepatitis B virus, HCV: Hepatitis C virus, HVPG: Hepatic venous pressure gradient, LCP: Patients with concomitant liver cirrhosis, MELD: Model of end stage liver disease, TIPS: Transjugular intrahepatic portosystemic shunt.

### Morbidity and mortality

Postoperative outcome was drastically worse for LCP with significantly higher morbidity (p = 0.035, Table 3) and 30-day and 90-day mortality rates (p<0.001). Renal failure (p = 0.020), anastomotic leakages (p = 0.002), and sepsis (p = 0.006) were significantly more frequent in LCP, whereas pulmonary complications (p = 0.854) and hepatic failure (p = 0.095) were equally distributed in both groups. Of 13 LCP with severe complications Clavien Dindo ≥ 3, 8 (61.5%) underwent surgical revision.

**Table 3:** Unadjusted analysis of postoperative outcomes by severity of liver cirrhosis.

	NLCP	LCP		LCP		LCP		
			p-value	MELD ≤ 9	p-value	MELD >9	p-value	p-value
	n = 156	n = 14	Non-LC vs. LC	n = 9	NLCP vs. MELD ≤ 9	n = 5	NLCP vs. MELD >9	MELD ≤ 9 vs. MELD >9
30-d mortality	12 (7.7)	8 (57.1)	**<0.001**	5 (55.6)	**0.001**	3 (60.0)	**0.006**	1.000
90-d mortality	15 (9.6)	9 (64.3)	**<0.001**	6 (66.7)	**<0.001**	3 (60.0)	**0.01**	0.622
Pulmonary complication (yes)	39 (25.0)	6 (42.9)	0.203	4 (55.6)	0.241	2 (40.0)	0.602	1.000
Cardiac complication (yes)	13 (8.3)	1 (7.1)	0.872	0 (0.0)	0.618	1 (20.0)	0.371	0.357
Renal failure (yes)	24 (15.4)	6 (42.9)	**0.020**	3 (33.3)	0.166	3 (60.0)	**0.034**	0.580
Hepatic failure (yes)	11 (7.1)	3 (21.4)	0.095	2 (22.2)	0.152	1 (20.0)	0.326	1.000
Anastomotic Leakage (yes)	35 (22.4)	9 (64.3)	**0.002**	5 (55.6)	**0.039**	4 (80.0)	**0.013**	0.580
Chyle leak (yes)	5 (3.2)	1 (7.1)	0.408	0 (0.0)	1.000	1 (20.0)	0.175	0.357
Sepsis (yes)	33 (21.5)	8 (57.1)	**0.006**	5 (55.6)	**0.031**	3 (60.0)	0.074	1.000

All numbers are indicated as absolute numbers and percentages or mean ± standard deviation. p-values in bold indicate statistical significance between cohorts. d: Days, LCP: Patients with concomitant liver cirrhosis, MELD: Model for End-Stage Liver Disease score, n: Number, NLCP: Patients without concomitant liver cirrhosis.

By subdividing LCP according to MELD score (Low-MELD ≤ 9, High-MELD > 9), both groups showed comparable results in contrast to NLCP, especially in terms of mortality. While sepsis and renal failure revealed only significant differences between NLCP and the High-MELD group, sepsis only differed significantly between NLCP and the Low-MELD group. Interestingly, postoperative outcomes of the Low-MELD and High-MELD groups were equal with no significant difference. After adjustment for gender, age, surgical approach and comorbidity index, LCP demonstrated 10.5 times higher odds for 30-day ([95%CI 2.704–40.763], p = 0.001; Table 4) and 16.5 times higher odds for 90-day mortality ([95%CI 3.873–70.014], p<0.001) compared to NLCP. Moreover, increased risks for renal failure (p = 0.010), anastomotic leakage (p = 0.020), and sepsis (p = 0.015) are shown for cirrhotic patients.

**Table 4:** Risk-adjusted analysis of postoperative outcomes of cirrhotic and non-cirrhotic patients.

	LCP vs. NLCP (OR [95%CI])	Regression coefficient of variable	Regression coefficient of constant	p-value
30-d mortality	10.499 [2.704–40.763]	2.351	0.674	**0.001**
90-d mortality	16.466 [3.873–70.014]	2.801	1.939	**<0.001**
Major complications	3.536 [0.682–18.344]	1.263	-0.419	0.133
Pulmonary complication (yes)	2.529 [0.702–9.118]	0.928	2.346	0.156
Cardiac complication (yes)	1.274 [0.120–13.495]	0.242	24.625	0.138
Renal failure (yes)	6.216 [1.562–24.730]	1.827	2.358	**0.010**
Hepatic failure (yes)	2.990 [0.584–15.314]	1.095	3.98	0.189
Anastomotic Leakage (yes)	4.412 [1.262–15.421]	1.484	-0.915	**0.020**
Chyle leak (yes)	1.290 [0.107–15.512]	0.255	2.602	0.841
Sepsis (yes)	4.765 [1.353–16.778]	1.561	1.591	**0.015**
Long-term survival (m) ^a^	2.45 e^-11^[2.089 e^-19^–0.003|]	-24.429	61.737	**0.010**

Generalized linear model, adjusted for gender, age, surgical approach and comorbidities by Charlson Comorbidity Index. p-values in bold indicate statistical significance between cohorts. ^a^ exclusion 19.2% of NLCP lost to follow-up. CI: Confidence interval, d: Days, m: Month, OR: Odds ratio.

### Prediction of mortality

The characteristics of LCP with and without mortality within 30 and 90 days after surgery are depicted in Table 5. Univariate analysis revealed significant differences for tumor stages (p = 0.036), presence of preoperative ascites (p = 0.031, p = 0.038) and portal hypertension (p = 0.031, p = 0.038). MELD and Child Score just as their categories failed to predict 30-day or 90-day mortality. Hence, several components of these scores correlated significantly with mortality, such as bilirubin (p = 0.018, p = 0.001) and INR (p = 0.009, p = 0.002), and also platelet count (p = 0.002, p = 0.003) demonstrated statistical significance. Moreover, the occurrence of specific complications was not associated with mortality.

**Table 5:** Univariate analysis of 30-day and 90-day Mortality Rates of cirrhotic patients.

	30-d Mortality			90-d Mortality		
	no	yes	p-value	no	yes	p-value
	n = 12	n = 13		n = 8	n = 17	
Gender						
male	3 (50.0)	4 (50.0)	1.000	2 (40.0)	5 (55.6)	1.000
female	3 (50.0)	4 (50.0)		3 (60.0)	4 (44.4)	
Age (y)	61.3±8.4	66.1±8.9	0.326	62.8±8.4	64.8±9.2	0.700
Charlson Comorbidity Index	3.3±1.8	4.3±1.3	0.279	3.2±1.9	4.2±1.2	0.240
Histopathology						
AC	2 (33.3)	4 (50.0)	0.627	1 (20.0)	5 (55.6)	0.301
SCC	4 (66.7)	4 (50.0)		4 (80.0)	4 (44.4)	
pT						
no tumor	2 (33.3)	0 (0.0)	**0.036**	2 (40.0)	0 (0.0)	0.058
1	4 (66.7)	2 (25.0)		3 (60.0)	3 (33.3)	
2	0 (0.0)	2 (25.0)		0 (0.0)	2 (22.2)	
3	0 (0.0)	4 (50.0)		0 (0.0)	4 (44.4)	
4	0 (0.0)	0 (0.0)		0 (0.0)		
Neoadjuvant Therapy	2 (33.3)	4 (50.0)	0.627	3 (60.0)	5 (55.6)	0.872
Procedure						
Thoracoabdominal (Ivor-Lewis)	4 (66.7)	7 (87.5)	0.538	3 (40.0)	8 (88.9)	0.505
Cervico-thoracoabdominal (McKeown)	2 (33.3)	1 (12.5)		2 (40.0)	1 (11.1)	
Approach						
Open	6 (100.0)	5 (62.5)	0.309	3 (60.0)	8 (88.9)	0.207
MIC	0 (0.0)	3 (37.5)		2 (40.0)	1 (11.1)	
LC preop diagnosed	2 (33.3)	5 (62.5)	0.592	1 (20.0)	6 (66.7)	0.266
Ascites praeop	0 (0.0)	5 (62.5)	**0.031**	0 (0.0)	5 (55.6)	**0.038**
Esophageal varices	2 (33.3)	4 (50.0)	0.627	1 (20.0)	5 (55.6)	0.301
Portal hypertension	0 (0.0)	5 (62.5)	**0.031**	0 (0.0)	5 (55.6)	**0.038**
Etiology						
Ethyl toxic	6 (100.0)	6 (75.0)	0.417	5 (100.0)	7 (77.8)	0.523
HBV/HCV	0 (0.0)	1 (12.5)		0 (0.0)	1 (11.1)	
Unclear	0 (0.0)	1 (12.5)		0 (0.0)	1 (11.1)	
Pulmonary complications	2 (33.3)	4 (50.0)	0.627	2 (40.0)	4 (44.4)	1.000
Cardiac Complications	0 (0.0)	1(12.5)	1.000	0 (0.0)	1 (11.1)	1.000
Ascites postoperative	0 (0.0)	1 (12.5)	1.000	0 (0.0)	1 (11.1)	1.000
Renal Failure	1 (16.7)	5 (62.5)	0.138	1 (20.0)	5 (55.6)	0.198
Hepatic Failure	0 (0.0)	3 (37.5)	0.209	0 (0.0)	3 (33.3)	0.258
Anastomotic Leakage	4 (66.7)	5 (62.5)	1.000	3 (60.0)	6 (66.7)	0.803
Chyle leak	1 (16.7)	0 (0.0)	0.231	1 (20.0)	0 (0.0)	0.357
Sepsis	2 (33.3)	6 (75.0)	0.277	2 (40.0)	6 (66.7)	0.580
MELD-Score	8.5±2.6	10.3±4.0	0.398	8.6±2.9	10.0±3.9	0.198
MELD-Score						
≤ 9	4 (66.7)	5 (62.5)	1.000	3 (60.0)	6 (66.7)	0.803
> 9	2 (33.3)	3 (37.5)		2 (40.0)	3 (33.3)	
CTP score	6.8±0.4	7.3±1.4	0.443	6.8±0.4	7.2±1.3	0.396
CTP						
A	1 (16.7)	3 (37.5)	0.580	1 (20.0)	3 (33.3)	0.597
B	5 (83.3)	5 (62.5)		4 (80.0)	6 (66.7)	
Hypalbuminaemia						
< 25mg/dl	1 (16.7)	2 (25.0)	1.000	1 (20.0)	2 (22.2)	1.000
≥ 25 mg/dl	5 (83.3)	6 (75.0)		4 (80.0)	7 (77.8)	
Bilirubin(mg/dl)	0.5±0.3	1.4±0.8	**0.018**	0.4±0.2	1.3±0.8	**0.001**
Creatinine (mg/dl)	1.1±0.5	0.9±0.3	0.438	1.1±0.6	0.9±0.3	0.615
INR (%)	1.0±0.0	1.3±0.3	**0.009**	1.0±0.0	1.2±0.3	**0.002**
Albumin (mg/l)	19.8±8.0	24.8±11.1	0.429	19.4±8.8	24.5±10.5	0.215
Platelets (Mrd/l)	334.5±118.8	127.3±78.8	**0.002**	351±124.9	141.1±86.0	**0.003**

All numbers are indicated as absolute numbers and percentages or mean ± standard deviation. p-values in bold indicate statical significance between cohorts. AC: Adenocarcinoma, d: Days, dl: Decilitre, HBV: Hepatitis B virus, HCV: Hepatitis C Virus, INR: International Normalized Ratio, l: Litre, MELD-Score: Model for End-Stage Liver Disease, mg: Milligram, Mrd: Milliard, SCC: Squamous cell cancer, UICC: Union internationale contre le cancer, y: Years.

**Fig 1:**
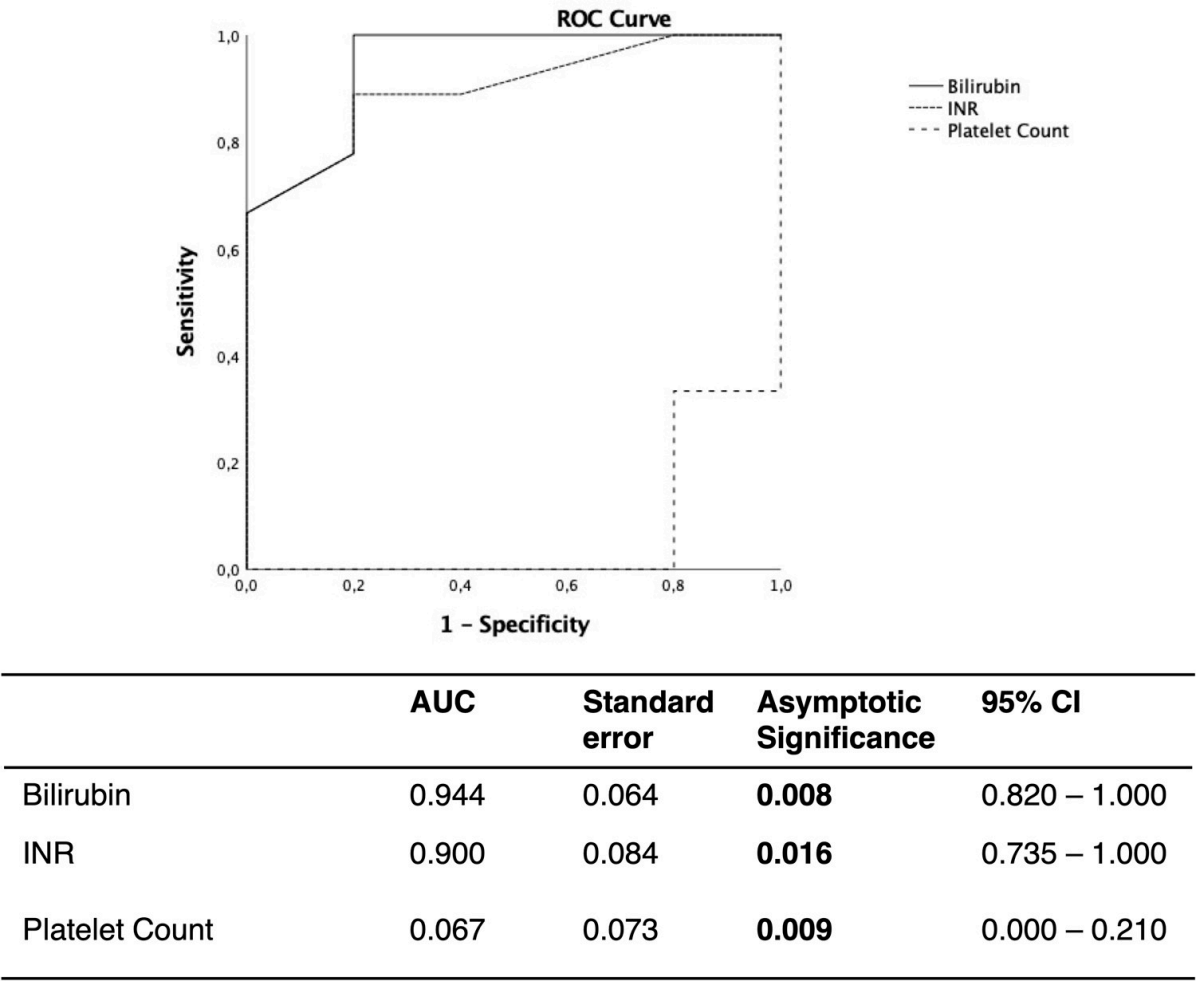
Comparison of receiver operating characteristic (ROC) curves for prediction of 90-day mortality in cirrhotic patients by Bilirubin, INR and platelet count. Bilirubin and platelet count providing an excellent diagnostic capacity. p-values in bold indicate statistical significance. AUC: Area under the curve, CI: Confidence interval, INR: International normalized ratio.

In ROC analysis, MELD score, Child Score and CCI failed in predicting 30-day or 90-day mortality (auROC = 0.644–0.688, S1 and S2 Figs). In contrast, bilirubin and INR proofed excellent prognostic capacity in predicting both, 30-day ad 90-day mortality (bilirubin: 30-d M auROC = 0.875 p = 0.020, 90-d M auROC = 0.944, p = 0.008; INR: 30-d M auROC = 90.6% p = 0.012, 90-d M auROC = 90.0 p = 0.016, Figs 1 and S3).

### Long-term outcome

**Fig 2:**
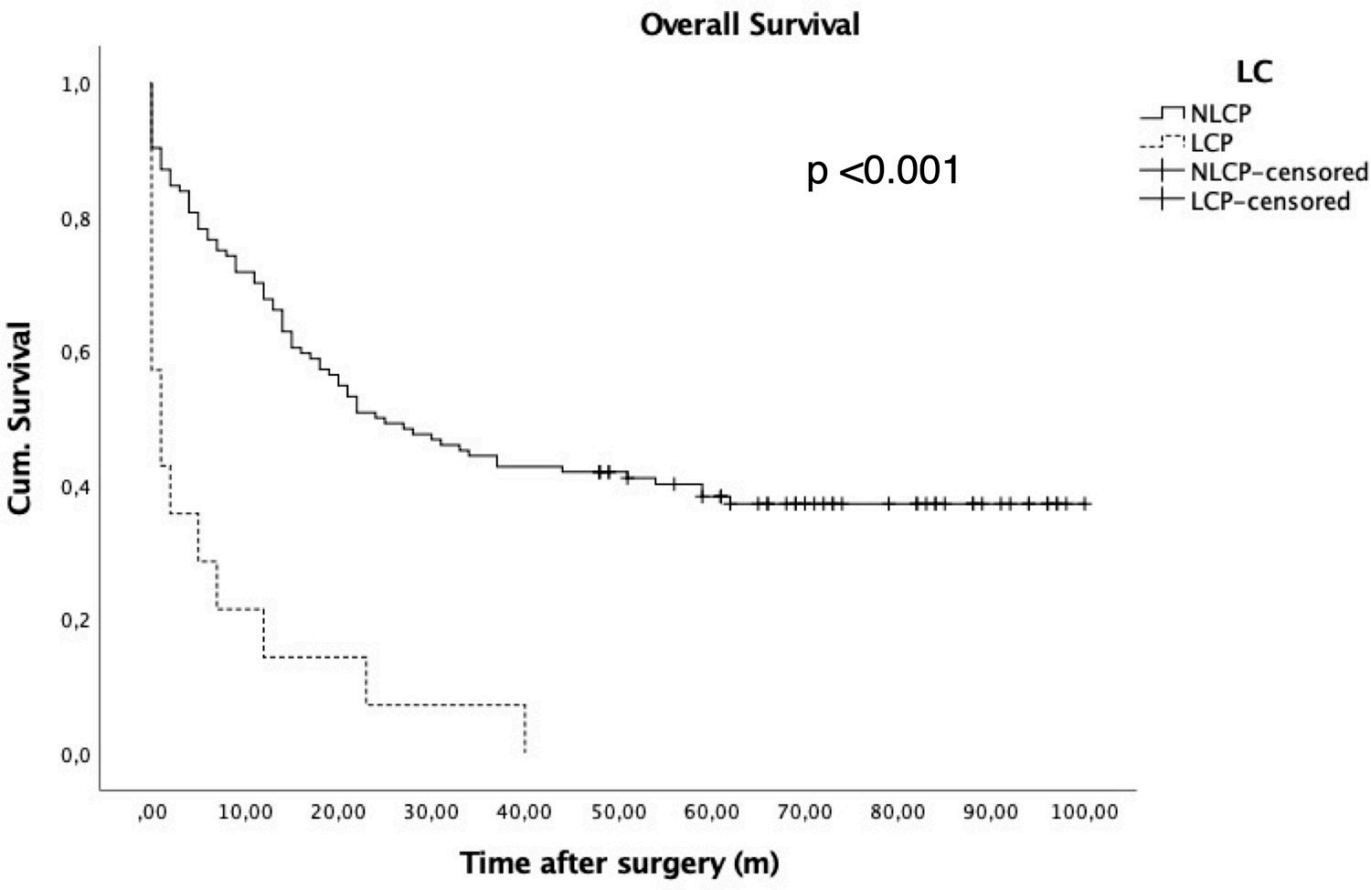
Cumulative Survival curves after esophagectomy of cirrhotic (LCP) and non-cirrhotic patients (NLCP). 1-, 3- and 5-year survival were 79.1%, 44.40% and 32.3% in the NLCP cohort and 21.4%, 7.1% and 0.0% in the LCP group. 19.2% of NLCP were lost to follow-up and excluded from analysis.

Cirrhotic patients had a significantly poorer prognosis compared to NCLP. After exclusion of 19.2% of NLCP, who were lost to follow up, 1-, 3- and 5-year survival for NLCP were 71.8%, 44.4% and 32.3% compared to 21.4%, 7.1% and 0.0% for LCP, respectively (p<0.001, Fig 2). Considering only patients, who were discharged alive, NLCP also demonstrated significantly better long-term survival (p = 0.023, S4 Fig).

## Discussion

This study demonstrates enormous perioperative morbidity and mortality for cirrhotic patients undergoing radical esophagectomy for esophageal cancer. Moreover, the disastrous long-term outcome of perioperative survivors questions the justification of these risks.

These findings are in line with previous meta-analysis demonstrating higher complication rates in LCP (39.7–83.3%) compared to non-cirrhotic patients [20,21]. As specific complications, anastomotic leakage rate, sepsis, and renal failure are significantly more frequent in this LCP cohort. Leakages rates of cirrhotic patients are reported to be more frequent [14,22] or more frequently associated with surgical death [14,15] compared to NLCP, while others describe comparable rates to the literature [20] or comparison group but more severe manifestations [18,21]. Impaired conduit perfusion by aggravated venous outflow after the division of coronary veins has been discussed as a possible factor. Therefore, preoperative TIPS might have a positive impact on selected patients [23]. Moreover, protein metabolism disorder and immune dysfunction might further affect anastomotic closure [24].

Sepsis is also significantly more frequent among LCP, associated with 75% of 30-day mortality in our cohort. The main contributing factor might be an acquired immune dysfunction syndrome of cirrhotic patients [15,18,25]. Furthermore, renal failure is more common among LCP compared to NLCP, which affects in particular patients of the High-MELD group significantly (Table 3). Septical conditions might contribute to acute renal failure, but since Low-MELD patients are equally concerned by sepsis in contrast to kidney dysfunction, a hepato-renal component is to be assumed. In literature, only one publication reports postoperative renal failure, finding a highly significant distribution towards LCP with no association to postoperative deaths [15]. In contrast to recent publications [14–16,18,21,22], postoperative ascites, pleural effusion, and postoperative liver failure are of unimportance in this LCP cohort. Potentially, the high proportion of intraoperatively diagnosed liver cirrhosis of 50%, which might be less affected by liver disease, as well as the high ratio of TIPS in preoperatively hydropic decompensated patients might contribute to this finding. Furthermore, although a higher rate of pulmonary complications was registered for LCP (25.0% vs. 42.9%, p = 0.203, Table 3), no significant difference to NLCP could be found, also after adjustment for gender, age, surgical approach, and comorbidities (Table 4). The small sample size of this LCP cohort could prevent this difference from becoming significant. Potentially, a minimally-invasive rate twice of the NLCP cohort might also contribute to a reduction of pulmonary complications, as suggested by recent publications [15,20]. The 30-day and 90-day mortality rates, accounting for 57.1% and 64.3%, respectively, precisely describe the strongly increased perioperative risk of cirrhotic patients, which highly significantly differ from NLCP (p<0.001) before and after adjustment for confounders. In literature, perioperative mortality is reported to be lower, reporting 10.8–25.0%, for in-hospital mortality with a minor subset of studies referring to 30-day or 90-day mortality. Moreover, published meta-analyses indicated a potential publication bias [20,21] or low to moderate confidence in estimates [22]. Hence, it must be at least assumed that the underlying evidence could be biased by underreporting and quality of reported outcomes.

This NLCP cohort demonstrates a high rate of 7.7% for 30-day mortality. Though, in contrast to the literature, this rate does not double after 90 days (9.6%) but normalizes to published rates for 90-day mortality (7.0–13.3% [26–28]). This finding might be addressed to an outstanding ratio of severe comorbidities in this NLCP cohort as indicated by CCI ≥ 3 (52.6%). In comparison, recent publications included a minor subset of patients with severe comorbidities (CCI ≥ 3: 1,4%, 30-d mortality 4.2% [26]). Therefore, 30-day mortality, which is discussed as an indicator for hospital’s capability to provide perioperative care and is decisively influenced by patients’ age, and comorbidities, might be poorer, whereas 90-day mortality, reflecting surgical and cancer management decisions, is within recently published ranges [26–28].

The chance of cure is drastically poorer for LCP compared to NLCP: in 5-year survival analysis, 7.1% of cirrhotic patients are alive 3 years post-surgery. Even after the exclusion of postoperative deaths, survival of the LCP is still significantly worse (p = 0.023). The limited available evidence of three studies reporting on this subject shows a contradictory picture: two studies cannot find a difference in long-term survival [16,18], while another proved the same after excluding postoperative deaths [14]. These data have been summarized in two meta-analyses both finding no significant differences for LCP and NCLP in terms of long-term survival. Nevertheless, both shed new light on the existing evidence: while one analysis reports high heterogeneity (I^2^ = 74.8%) suggesting a random effect [22], the other observes a tendency for unfavourable survival of LCP [21].

The risk assessment for LCP prior to esophagectomy remains elusive, as claimed by several studies [15,20,21]: in contrast to other publications [14,16,18,21], MELD and CTP score are of no predictive value in this cohort, but components of both scores as preoperative ascites, bilirubin levels, and INR prove good prognostic capacity. Therefore, patients with completely normal bilirubin and INR levels and without any current or former sign of portal hypertension or hydropic decompensation could potentially be evaluated as candidates for curative esophagectomy.

Not only the retrospective nature of this study–although the data are derived from a prospectively maintained database—restrains this research but also an inherent selection bias. Therefore, only CTP A and B patients were included, as recommended by the present evidence, and further unmeasurable factors may have led to the reluctance of responsible surgeons to operate on these fragile patients. Further, only a limited number of 14 LCP could be identified from the database and limit the significance of these findings. Thus, reported observations need to be interpreted with caution. Further studies are needed to evaluate these findings.

Overall, LC remains a crucial risk factor for major surgery, thus, for esophagectomy. A careful patient selection should be mandatory, which might approve patients for curative surgery with completely normal bilirubin and INR levels and without any current or former sign of portal hypertension or hydropic decompensation. Though, associated additionally with worse long-term survival, the justification for curative esophagectomy for cirrhotic patients remains questionable and needs further research.

## Supporting information

S1 Checklist (DOCX)

S1 Fig Comparison of receiver operating characteristic (ROC) curves for prediction of 30-day mortality in cirrhotic patients by MELD score, Child Score and Charlson Comorbidity Index. All scores fail in prediction of 30-day mortality. AUC: area under the curve, CI: confidence interval.
(TIF)

S2 Fig Comparison of receiver operating characteristic (ROC) curves for prediction of 90-day mortality in cirrhotic patients by MELD score, Child Score and Charlson Comorbidity Index. All scores fail in prediction of 30-day mortality. AUC: area under the curve, CI: confidence interval.
(TIF)

S3 Fig Comparison of receiver operating characteristic (ROC) curves for prediction of 30-day mortality in cirrhotic patients by Bilirubin, INR and platelet count. Bilirubin provides an excellent and INR an outstanding diagnostic capacity, whereas the platelet count fails in prediction of 30-day mortality. p-values in bold indicate statistical significance between cohorts. AUC—area under the curve, CI—confidence interval, INR—international normalized ratio.
(TIF)

S4 Fig Cumulative Survival curves after esophagectomy of cirrhotic (LCP) and non-cirrhotic patients (NLCP). 1-, 3- and 5-year survival were 79.1%, 50.0% and 36.4% in the NLCP cohort and 60.0%, 7.1% and 20.0% in the LCP group. In-hospital deaths and 19.2% of NLCP were lost to follow-up and excluded from analysis. p-values in bold indicate statistical significance between cohorts.
(TIF)

S1 Data (XLSX)
