# Supplementary figures and images for: Risk stratification of cirrhotic patients undergoing esophagectomy for esophageal cancer: A single-centre experience
Source: PLoS One. 2022 Mar 9;17(3):e0265093. doi: 10.1371/journal.pone.0265093 (PMC8906633; doi:10.1371/journal.pone.0265093)

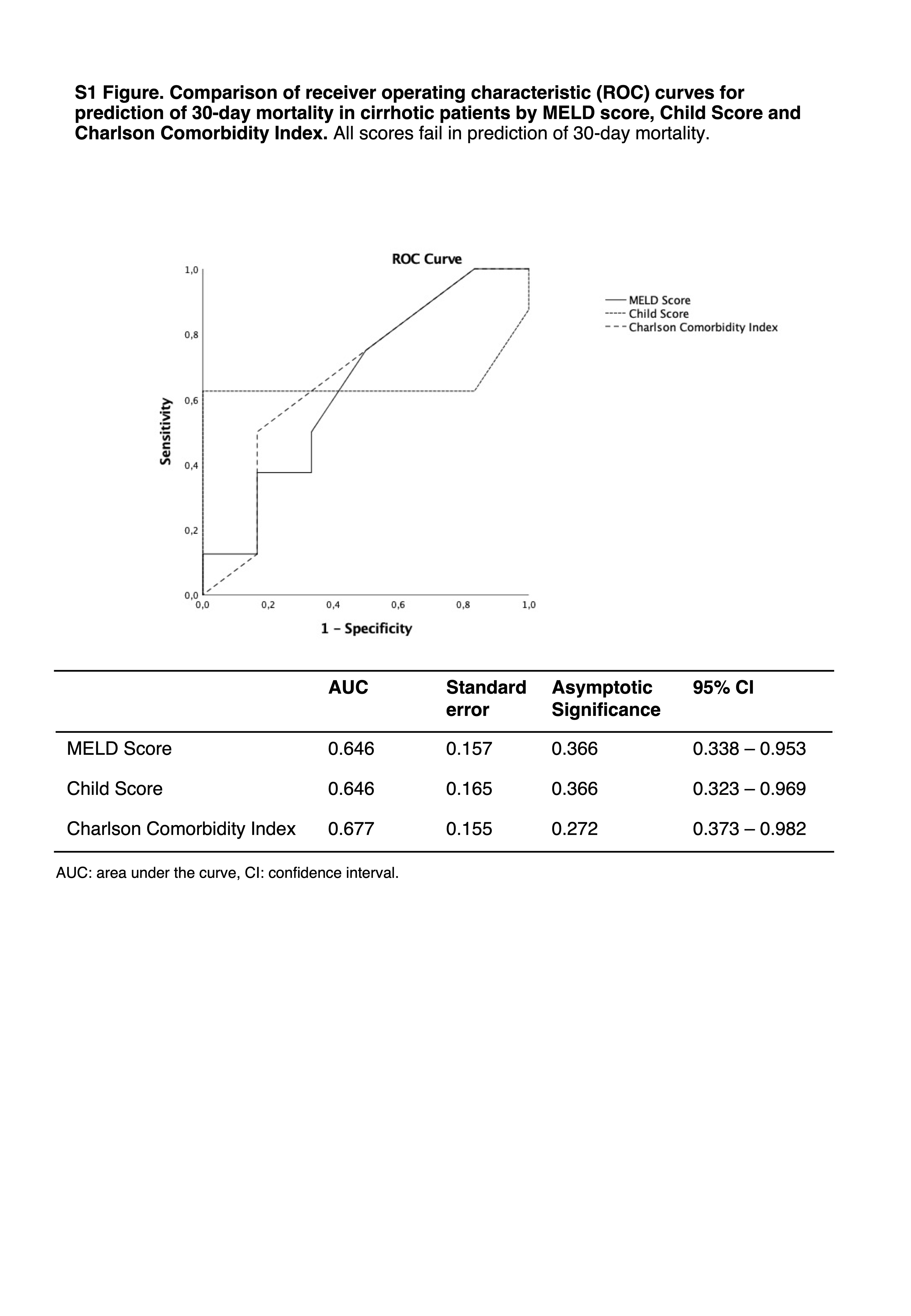

Supplement: S1 Fig — All scores fail in prediction of 30-day mortality. AUC: area under the curve, CI: confidence interval. (TIF) [file pone.0265093.s002.tif]

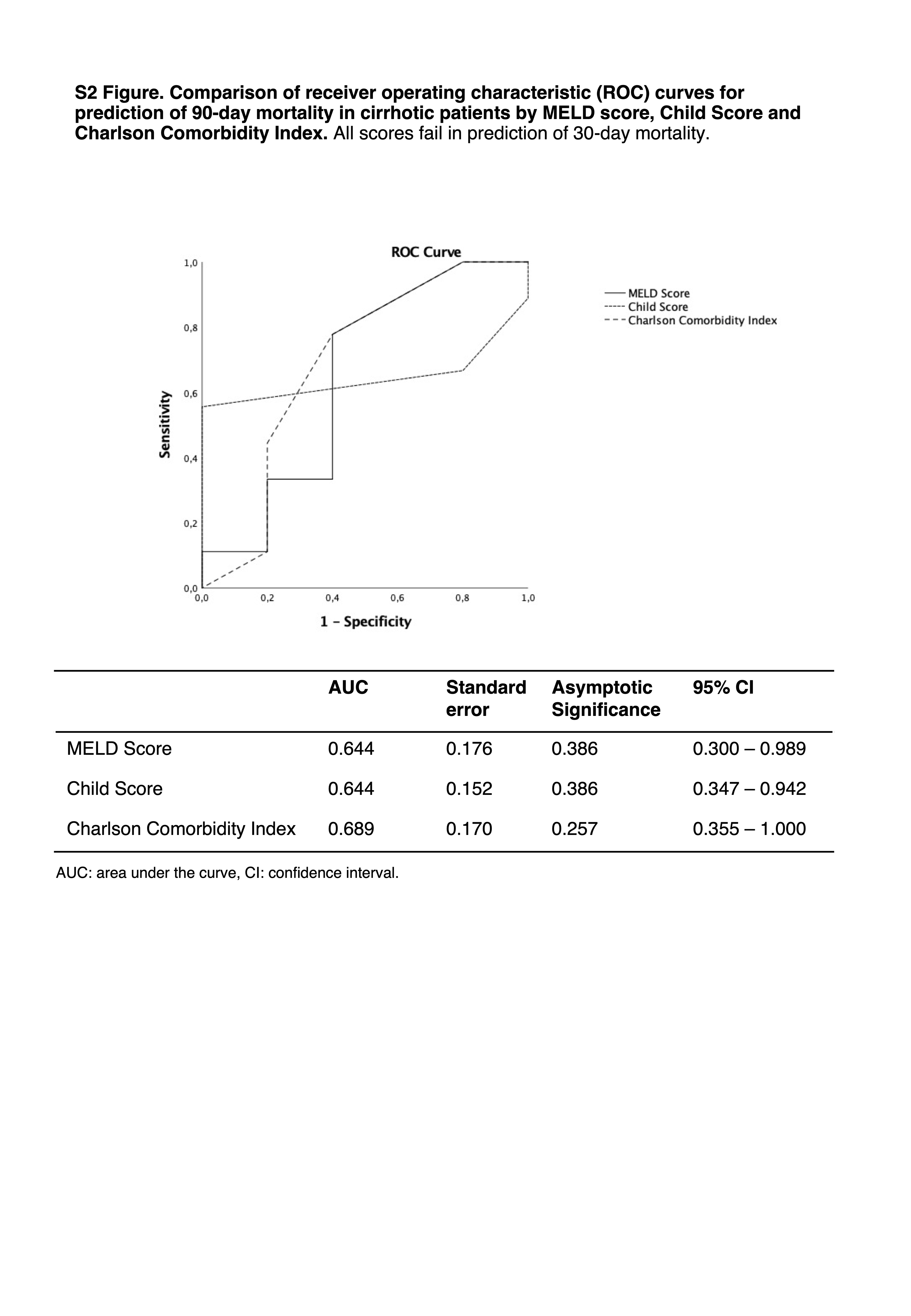

Supplement: S2 Fig — All scores fail in prediction of 30-day mortality. AUC: area under the curve, CI: confidence interval. (TIF) [file pone.0265093.s003.tif]

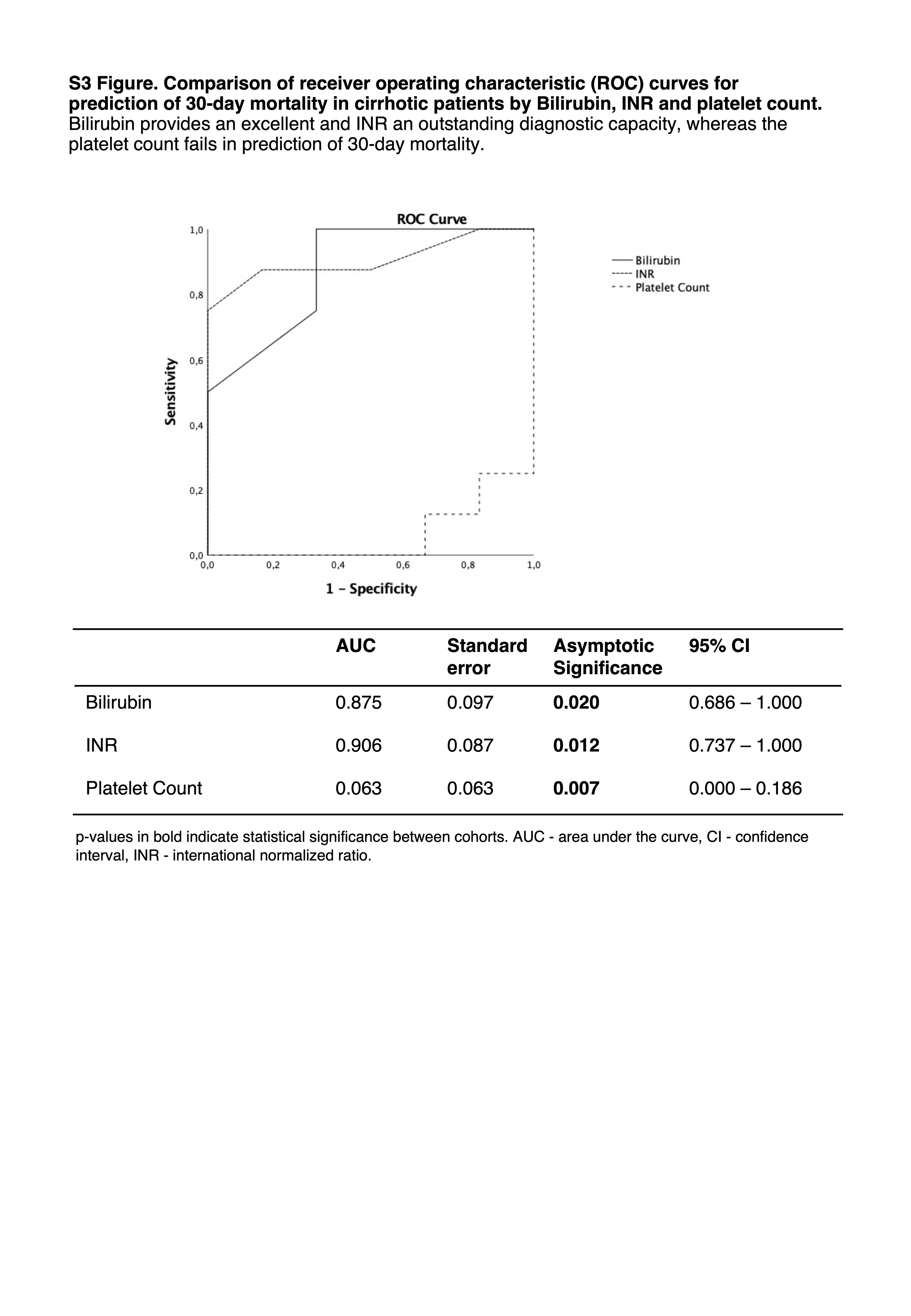

Supplement: S3 Fig — Bilirubin provides an excellent and INR an outstanding diagnostic capacity, whereas the platelet count fails in prediction of 30-day mortality. p-values in bold indicate statistical significance between cohorts. AUC—area under the curve, CI—confidence interval, INR—international normalized ratio. (TIF) [file pone.0265093.s004.tif]

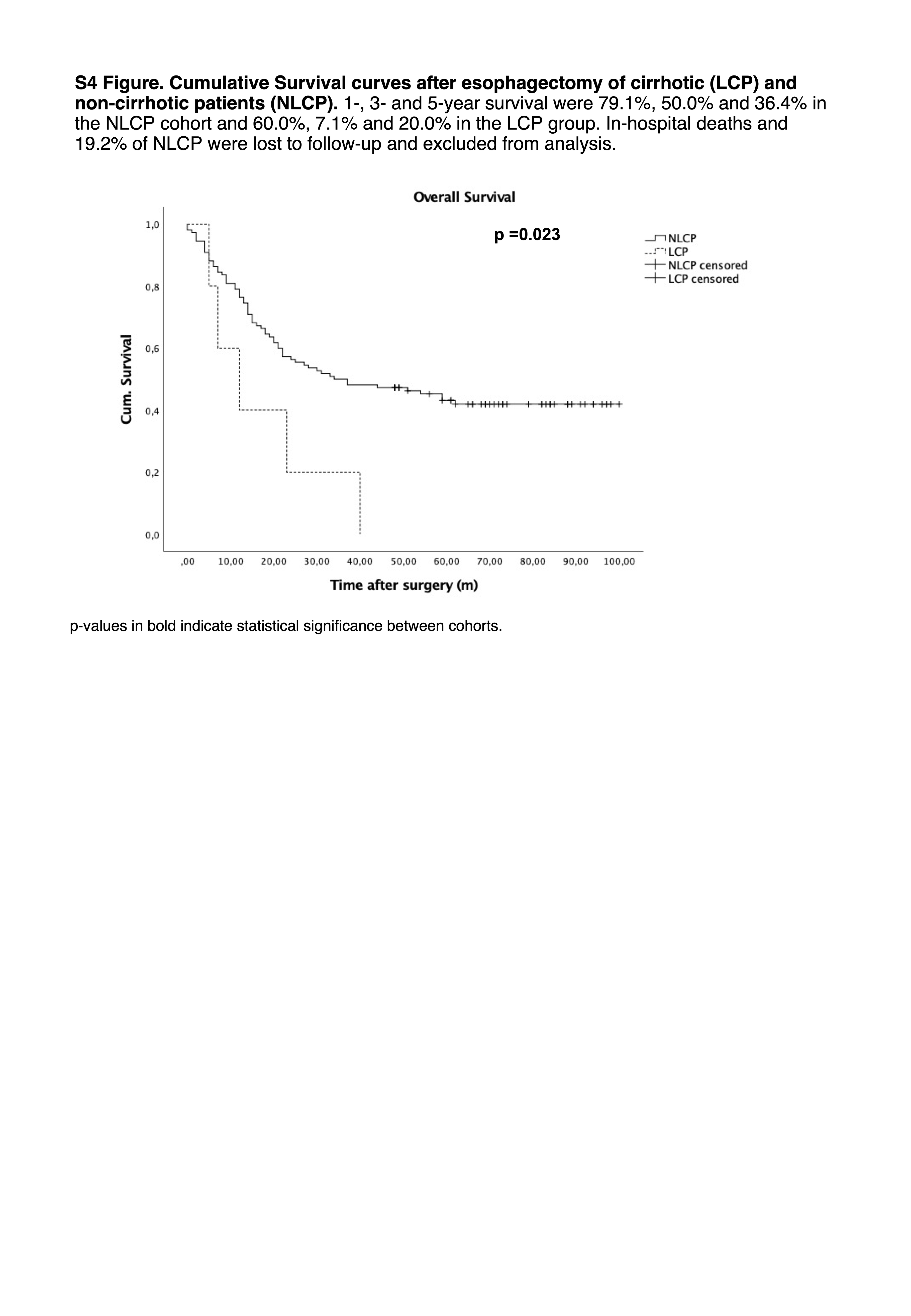

Supplement: S4 Fig — 1-, 3- and 5-year survival were 79.1%, 50.0% and 36.4% in the NLCP cohort and 60.0%, 7.1% and 20.0% in the LCP group. In-hospital deaths and 19.2% of NLCP were lost to follow-up and excluded from analysis. p-values in bold indicate statistical significance between cohorts. (TIF) [file pone.0265093.s005.tif]
